# Transcriptome Analysis of Salt Stress in *Hibiscus hamabo* Sieb. et Zucc Based on Pacbio Full-Length Transcriptome Sequencing

**DOI:** 10.3390/ijms23010138

**Published:** 2021-12-23

**Authors:** Longjie Ni, Zhiquan Wang, Xiangdong Liu, Shuting Wu, Jianfeng Hua, Yunlong Yin, Huogen Li, Chunsun Gu

**Affiliations:** 1 Institute of Botany, Jiangsu Province and Chinese Academy of Sciences, Nanjing 210014, China; LongJieNi@njfu.edu.cn (L.N.); wangzhiquan@cnbg.net (Z.W.); 15939918977@163.com (X.L.); Salvia_Wu@163.com (S.W.); jfhua@cnbg.net (J.H.); ylyin@cnbg.net (Y.Y.); 2 College of Forest Sciences, Nanjing Forestry University, Nanjing 210037, China; hgli@njfu.edu.cn; 3 Jiangsu Key Laboratory for the Research and Utilization of Plant Resources, Jiangsu Provincial Platform for Conservation and Utilization of Agricultural Germplasm, Nanjing 210014, China

**Keywords:** full-length transcriptome, salt stress, WRKY transcription factor, semi-mangrove plant, *Hibiscus hamabo* Sieb. et Zucc

## Abstract

*Hibiscus hamabo* Sieb. et Zucc is an important semi-mangrove plant with great morphological features and strong salt resistance. In this study, by combining single molecule real time and next-generation sequencing technologies, we explored the transcriptomic changes in the roots of salt stressed *H. hamabo*. A total of 94,562 unigenes were obtained by clustering the same isoforms using the PacBio RSII platform, and 2269 differentially expressed genes were obtained under salt stress using the Illumina platform. There were 519 differentially expressed genes co-expressed at each treatment time point under salt stress, and these genes were found to be enriched in ion signal transduction and plant hormone signal transduction. We used *Arabidopsis thaliana* (L.) Heynh. transformation to confirm the function of the *HhWRKY79* gene and discovered that overexpression enhanced salt tolerance. The full-length transcripts generated in this study provide a full characterization of the transcriptome of *H. hamabo* and may be useful in mining new salt stress-related genes specific to this species, while facilitating the understanding of the salt tolerance mechanisms.

## 1. Introduction

Salt stress is a significant environmental stressor and one of the primary factors affecting crop development and output [1]. This problem has been aggravated by the recent increase in human activity and the occurrence of regular harsh weather [2]. As a result, understanding and revealing the mechanisms by which plants respond to salt stress is critical to the advancement of agricultural productivity, as well as plant and ecological environmental conservation [3,4]. Salinity stress causes osmotic stress, ion toxicity, and oxidative stress, leading to cell dehydration, biochemical process disruption, growth limitation and even the death of the whole plant [5]. The complex responses of plants to salinity stress include signal transduction, ion homeostasis, reactive oxygen species (ROS)-scavenging, and any other growth regulatory pathways [6]. Specific transcription factors and related genes involved in the salinity stress response are activated in the process [7]. However, the adaptive salt tolerance mechanisms may differ among different plant species.

Semi-mangrove plants are woody, amphibious plants that may grow in both intertidal and non-saline soils [8,9]. They have important ecological functions and have a remarkable capacity to adapt to different habitats [10]. *Hibiscus hamabo* Sieb. et Zucc, which is native to China, is an important semi-mangrove plant [11]. It is commonly grown in parks, marshes, and coastal beaches due to its outstanding qualities such as tolerance to salt, flooding, and barren soil [12]. Therefore, it provides an excellent resource for learning about how woody plants respond to salt stress [13]. *H. hamabo* has been proven in physiological tests to have high active oxygen scavenging and osmotic adjustment abilities under NaCl stress, as well as high salt tolerance [14]. In addition, our team investigated the *WRKY* and *bHLH* gene families of *H. hamabo* from a molecular biology standpoint and conducted functional research on associated genes [15,16]. However, the few available reports do not provide enough information on the mechanisms that underlie *H. hamabo*’s response to salt stress. Therefore, it is necessary to conduct a full-length transcriptome sequencing study on hibiscus.

The genome and transcriptome sequencing of mangrove plants and halophytes is crucial to understanding plant salt tolerance mechanisms [17,18,19]. The regulatory mechanism of *H. hamabo* under salt stress was discovered using next-generation sequencing (NGS) technology [20]. However, the accuracy of transcriptome is greatly reduced due to the evident shortcomings of second-generation sequencing, such as the short length of sequencing runs and poor assembly findings. The PacBio RSII third-generation sequencing technology can effectively overcome these problems and directly generate full-length transcripts. This offers greater advantages in constructing a complete transcriptome for any species without a reference genome, but due to the low sequencing depth, NGS data are still needed for auxiliary correction [21].

Therefore, based on these two sequencing technologies, we used PacBio sequencing technology to construct the full-length reference transcriptome for *H. hamabo* tissue for the first time. According to the RNA-sequencing (RNA-seq) data obtained by the Illumina sequencing platform, the transcriptional expression level under NaCl stress was corrected, compared, and analyzed. As the long-term treatments resembled the condition of real habitat of *H. hamabo*, longer times (48 h and 72 h), compared with previous work, treated samples were selected to predict resistance-forming of *H. hamabo* in response to salt stress in this study [20]. *HhWRKY79* was discovered based on the level of expression, and the function of *HhWRKY79* in regulating salt stress tolerance was preliminarily verified using transgenic *Arabidopsis thaliana* (L.) Heynh. (thaliana). These studies can lay a foundation for discovering genes related to the salt tolerance and molecular breeding of *H. hamabo*.

## 2. Results

### 2.1. SMRT Sequencing, Data Processing, and Annotation

In this experiment, nine samples were mixed to generate an Iso-seq library. The full-length transcript data were obtained through the PacBio Sequel platform. Finally, approximately 9.92 G subread bases were generated, which were obtained from 177,383 complete FLNCs after filtering out incomplete CGs. After clustering the redundant sequences, 121,091 isoforms were obtained. Ultimately, 94,562 non-redundant unigenes were obtained by second-generation transcript data correction. These genes were 161 to 14,547 bp in length, with an average length of 4206 bp and ExN50 length of 3766 bp (Table 1). Based on benchmarking universal single-copy ortholog (BUSCO) analysis, approximately 324 (75.52%) of the 429 expected embryophyte genes were identified as complete (Supplementary Materials Table S1).

**Figure 1:**
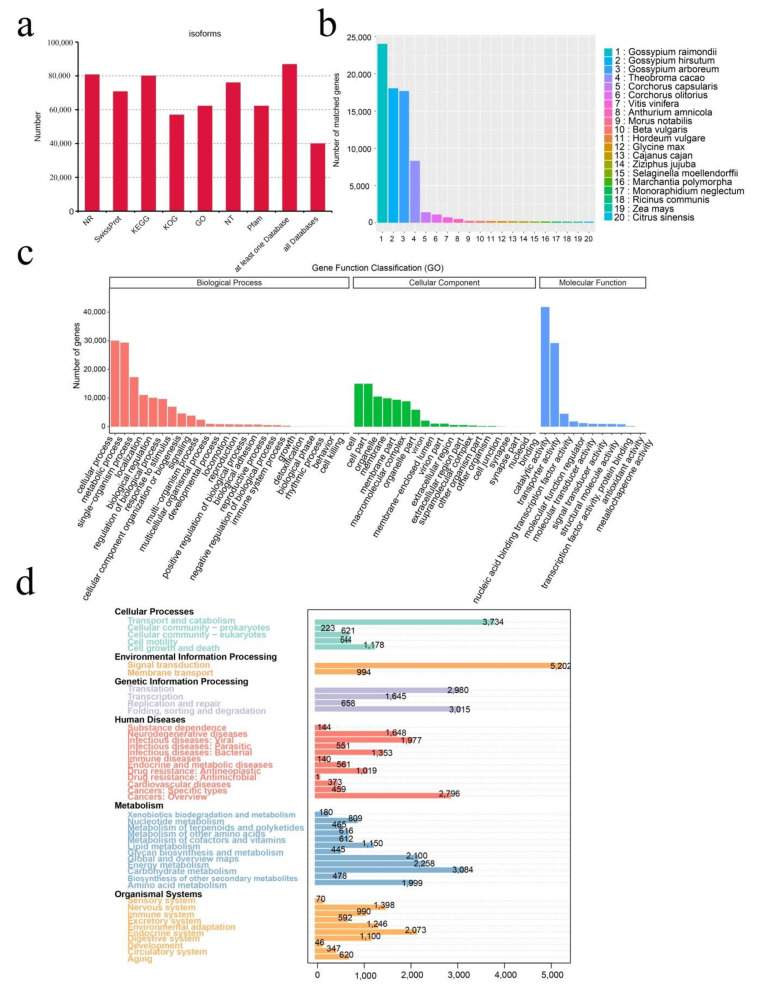
Iso-seq, assembly, and annotation of *H.* *hamabo.* (**a**) Statistical graph of the annotation results from seven databases. (**b**) The distribution of homologous species annotated in the NCBI non-redundant protein (NR) database and lengths of transcripts. (**c**) Gene Ontology (GO) classification of the assembled full-length transcripts. (**d**) Kyoto Encyclopedia of Genes and Genomes (KEGG) annotation of the assembled full-length transcripts.

In this study, 94,562 unigenes were functionally annotated in seven major databases (Figure 1a). The number of annotated transcripts ranged from 56,895 (60.12%, KOG) to 80,698 (85.33%, NR) in these databases. There were 86,793 (91.78%) unigenes annotated in at least one database, and 39,958 (42.26%) annotated in all databases. Among them, 62,158 (65.73%) and 25,009 (26.45%) unigenes were annotated in the GO and KEGG databases, respectively (Figure 1c,d). Additionally, in the NR database, there were 80,698 (85.33%) unigene sequences well-matched to known genes in plants (Figure 1b). The top three were all *Hibiscus* plants, which belong to the same family as *H.* *hamabo*, namely *Gossypium raimondii* Ulbrich (25.41%), *Gossypium hirsutum* L. (19.10%), and *Gossypium arboretum* L. (18.71%).

### 2.2. RNA-Seq Analysis during Salt Stress

**Figure 2:**
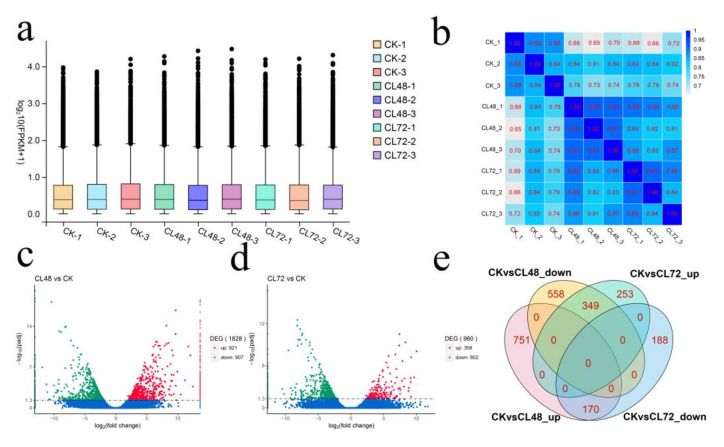
(**a**) Transcripts per million box plots. (**b**) Correlation analysis diagram of gene expression. (**c**) The M-versus-A (MA) plot of differentially expressed genes (DEGs) after 48 h of NaCl treatment. (**d**) The M-versus-A (MA) plot of DEGs after 72 h of NaCl treatment. (**e**) Venn diagram of all DEGs.

We constructed nine cDNA libraries from roots, which were treated with NaCI for 0 h (CK), 48 h (CL48) and 72 h (CL72). To obtain the expression level of each unigene, we used RSEM to compare the number of read counts for each gene in each sample to calculate the RSEM value. The box plot of RSEM values shows that the gene expression levels were unevenly distributed under different treatments (Figure 2a). The Pearson correlation coefficient indicated a high correlation between repeated samples, which can be used in follow-up research (Figure 2b). Differential expression analysis software DESeq2 was used to identify the DEGS (with padj < 0.05, |log_2_FoldChange| > 1). A total of 2269 DEGs, which participated in the salt stress response process, were obtained. Among them, compared with the CK, the numbers of DEGs between CL48 and CL72 were 1828 (921 up-regulated and 907 down-regulated) and 960 (358 up-regulated, 602 down-regulated), respectively (Figure 2c,d). In addition, there were 519 overlapping DEGs at 48 h and 72 h, as shown in the Venn (Figure 2e).

### 2.3. DEGs Cluster Analysis

**Figure 3:**
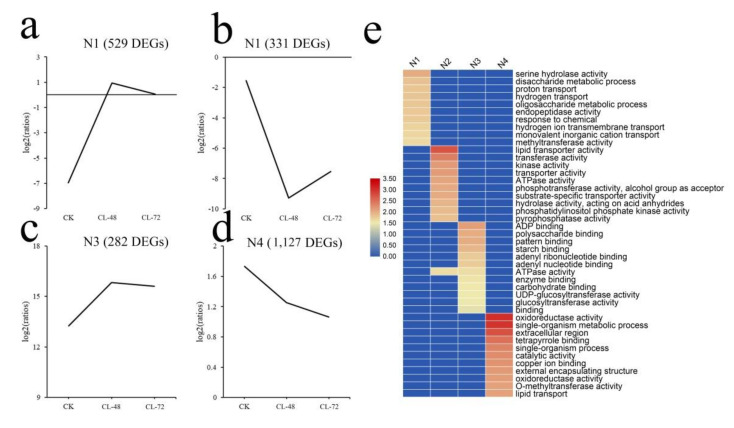
Clustering analysis of the DEGs. (**a**) The results for the N1 cluster. (**b**) The results for the N2 cluster. (**c**) The results for the N3 cluster. (**d**) The results for the N4 cluster. (**e**) Heat map of the GO annotations for each cluster.

We used the K-means algorithm to cluster genes with similar functions and finally divided them into four gene clusters: namely N1, N2, N3, and N4. These different gene clusters exhibited different expression patterns. The 529 genes in the N1 category were up-regulated after 48 h, and their expression levels began to decrease after 72 h; their GO annotations were mainly enriched in serine hydrolase activity and disaccharide metabolic process (Figure 3a). The 331 genes in the N2 category were significantly down-regulated before 48 h, after which their expression recovered and was eventually up-regulated; their GO annotations were mainly enriched in lipid transporter activity and transferase activity (Figure 3b). The 320 genes in the N3 category were upregulated continuously before 48 h and then began to be down-regulated; their GO annotations were mainly enriched in ADP binding and polysaccharide binding (Figure 3c). Finally, 1127 genes in the N4 category continued to be down-regulated after salt treatment and their GO annotations were mainly enriched in oxidoreductase activity and single-organism metabolic process. (Figure 3d).

### 2.4. Genes Encoding Transcription Factors in Response to Salt Stress

**Figure 4:**
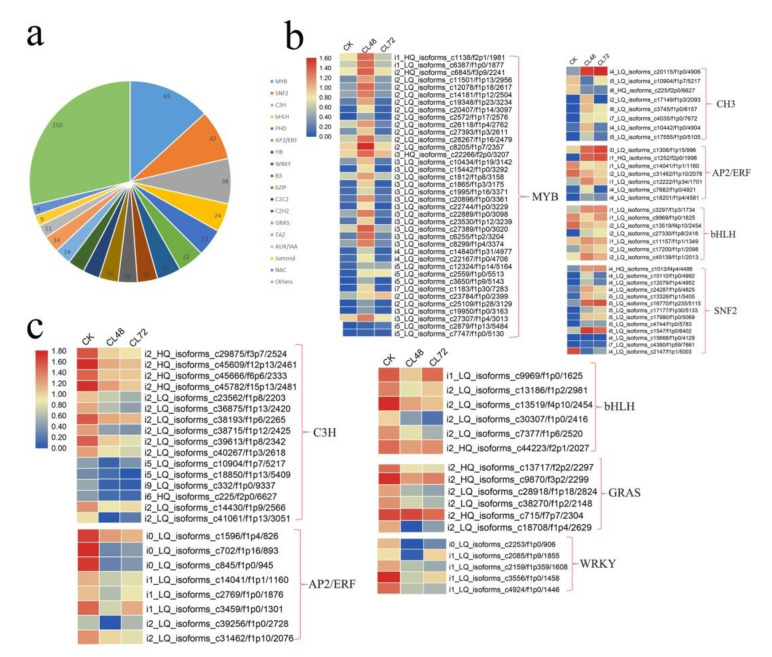
Transcription factor identification among the DEGs. (**a**) Pie chart of transcription factor statistics. (**b**) Expression heat map of the transcription factors from upregulated DEGs. (**c**) Expression heat map of the transcription factors from downregulated DEGs.

Transcription factor-mediated transcriptional regulation plays an important role in plant responses to abiotic stress. At least 522 genes were identified as transcription factors among the DEGs in *H. hamabo*. The top three families were MYB, SNF2, and C3H, with 69, 42 and 38 genes identified in each, respectively. Moreover, 22 and 17 AP2/ERF and WRKY genes, which are closely related to the salt stress response, were identified, respectively (Figure 4a). We conducted a classification study on these transcription factors. After counting the number of each gene family members, we found that MYB, SNF2, C3H, AP2/ERF, and bHLH accounted for the largest number of up-regulated transcription factors. Among the down-regulated transcription factors, C3H, AP2/ERF, bHLH, WRKY, and GRAS accounted for the largest number (Figure 4b). We then analyzed the expression patterns of the transcription factors between different treatments and found that some transcription factors had temporal and spatial specificity. For example, most of the up-regulated transcription factors increased significantly at 48 h and then began to decline (Figure 4c). Genes classified in Figure 4 were continuously downregulated under salt treatment.

### 2.5. Genes Related to Plant Hormones and Ion Signal Transduction Play an Important Role in the Response of H. Hamabo to Salt Stress

**Figure 5:**
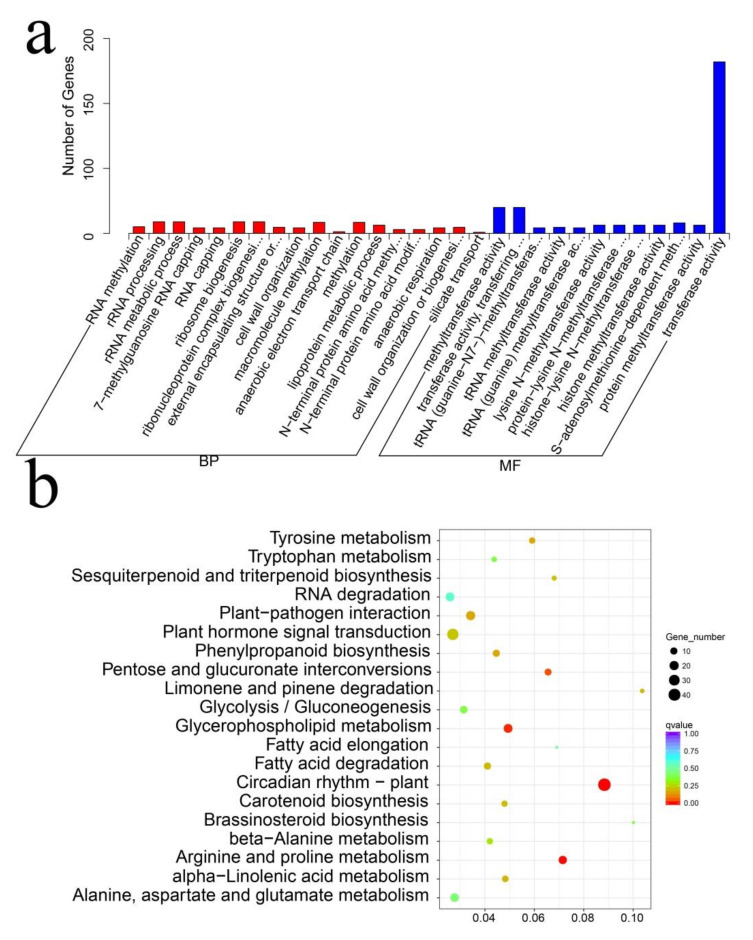
GO and KEGG analysis of DEGs from *H. hamabo* under salt stress. (**a**) GO analysis of DEGs from *H. hamabo* under salt stress. (**b**) KEGG analysis of DEGs from *H. hamabo* under salt stress.

To further study the defense mechanism of *H. hamabo* in response to salt stress, we carried out GO and KEGG annotation analyses of the 519 co-expressed DEGs. The top terms for molecular function was transferase activity, indicating that ion signal transduction plays a significant role in the response of *H. hamabo* to 48 h of salt stress (Figure 5a). Furthermore, the KEGG annotation analysis results showed that plant hormone signal transduction was also markedly enriched (Figure 5b), demonstrating that plant hormones also play an important role in the response to salt stress of *H. hamabo*.

**Figure 6:**
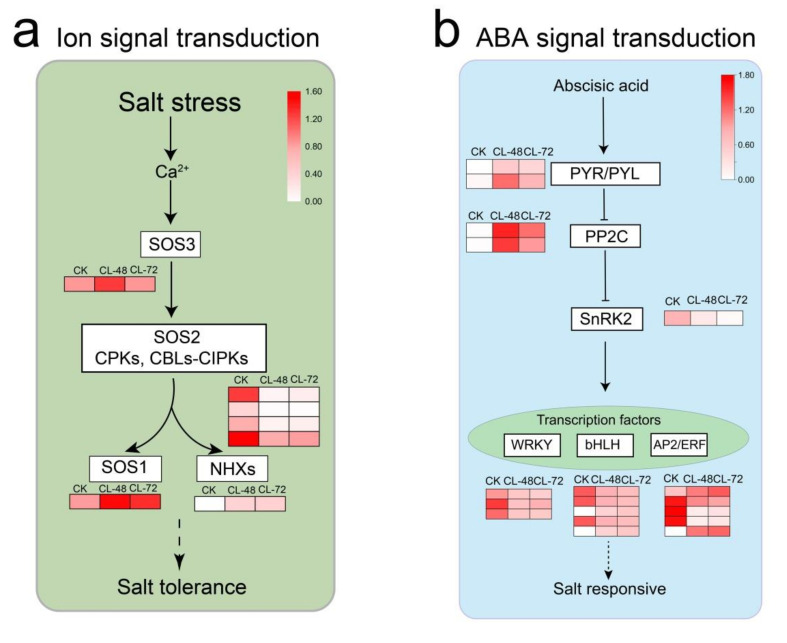
Genes involved in the response to salt stress in *H. hamabo* transcriptomes. Relative expression profiles were showed in white-red scale. ABA, abscisic acid; PYR/PYLs, pyrabactin resistance 1-like protein; PP2C, type 2C protein phosphatases; SnRKs, Snf1 (sucrose non-fermenting-1)-related protein kinases; CPK/CBL-CIPK, calcium-regulated phosphorylation systems; WRKY, WRKY transcription factor; bHlH, bHlH transcription factor; AP2, AP2/ERF transcription factor. Adapted with permission from Chen et al. [23]. Copyright 2021 Wiley Online Library, Zhu et al. [22]. Copyright 2016 Elsevier, Luo et al. [24].

Next, we mapped the 519 co-expressed DEGs into the abscisic acid (ABA) signal transduction and ion signal transduction regulatory networks and mapped the expression profiles of single genes involved in these two signal transduction processes based on previous study [22] (Supplementary Materials Table S2). Of the seven DEGs mapped to the ion signal transduction pathway, four were associated with SOS2, one with SOS3, one with SOS1, and one with NHX. It is worth noting that six DEGs were mapped to the ABA signal transduction pathways (Figure 6a). A total of four were up-regulated, which were associated two with PYLs/PYR and two with ABA co-receptor PP2C (Figure 6b). Two DEGs were down-regulated, which were associated with threonine/serine receptor kinase SnRK2 and ABF, respectively. In addition, 13 DEGs belonged to transcription factors that respond to stress, including three with WRKY, five with bHLH, and four with AP2/ERF.

### 2.6. HhWRKY79 Overexpression Increases Transgenic A. Thaliana Salt Tolerance

**Figure 7:**
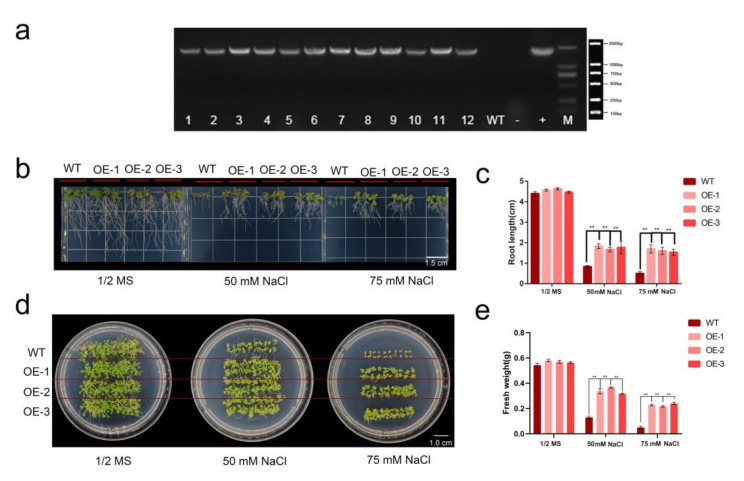
Genes involved in the response to salt stress in *H. hamabo* transcriptomes. Construction of *HhWRKY79* overexpression of *A. thaliana* plants and phenotypic changes under salt stress. (**a**) *HhWRKY79* overexpression *A. thaliana* gel map test results. M, 2000 bp marker; +, plasmid; −, empty vector; WT, wild-type *A. thaliana*; OE1-12, transgenic plants. (**b**) Comparison between root lengths in WT and transgenic *A. thaliana* under different concentrations of NaCl. (**c**) Statistic histogram of root length in WT and transgenic *A. thaliana* under different concentrations of NaCl. (**d**) Comparison of the growth of WT and transgenic *A. thaliana* under different concentrations of NaCl. (**e**) Statistical histogram of the fresh weight of WT and transgenic *A. thaliana* under different concentrations of NaCl. Significance was analyzed using Student’s *t*-test (** *p* < 0.01).

According to the results, we found that the i1_LQ_isoforms_c3556/f1p0/1458 in the *WRKY* transcription factor exhibited a high expression level and the expression level under salt treatment was always significantly lower than that under control. Therefore, we named it the *HhWRKY79* gene (NCBI accession number: MN972455) and used it for subsequent research. We constructed *HhWRKY79* overexpressing plants, obtained 12 T3 generation transgenic plants through resistance screening, and detected the expression of *HhWRKY79* in the T3 generation plants by PCR (Figure 7a). The results showed that *HhWRKY79* overexpressing *A. thaliana* plants were successfully constructed, and three independent transgenic lines, OE1, OE2 and OE3, were selected for follow-up experiments. Next, we grew WT and transgenic plants on 1/2 MS medium with different salt concentrations. After 10 days of growth, there were no significant differences in the root length and fresh weight between WT and transgenic plants grown on a normal 1/2 MS medium. As the salt concentration continued to increase, although the root length and fresh weight of the WT and transgenic plants decreased consistently, the decline in transgenic plants was slower than that in the WT plants. Under 50 mM NaCl, the root length of the WT plants was reduced by 81.82% and the fresh weight was reduced by 76.08%, while the root length of the transgenic plants was reduced by 60.45–64.02% and the fresh weight was reduced by 35.67–43.79%. When the NaCl concentration was increased to 75 mM, the root length of the WT plants was reduced by 88.64% and the fresh weight was reduced by 90.80%, while the root length of the transgenic plants was reduced by 64.33–65.67% and the fresh weight was reduced by 57.40–61.99%. These results indicated that the transgenic *A. thaliana* had a higher tolerance to salt stress than the WT, indicating that *HhWRKY79* can improve salt tolerance in *A. thaliana*.

## 3. Discussion

In this study, 86,793 (91.78%) of 94,562 unigenes were successfully annotated into seven databases; this is a higher percentage of annotated genes than in other plants, such as *Campeiostachys nutans* (Griseb.) J. L. Yang, B. R. Baum et C. Yen (78.01%) [25], *Vicia sativa* L. (66.10%) [26], and *Elymus sibiricus* L. (79.81%) [27]. Previously, our team used second-generation sequencing to acquire 75,257 unigene in *H. hamabo* [20]. We obtained a larger number of unigenes in our study by sequencing the entire transcriptome, and the unigenes are longer and more complete (4917 bp). We also employed the Illumina platform to look at gene expression in the roots of hibiscus after 48-h and 72-h of NaCl treatment. We found a total of 2269 DEGs at different NaCl treatment time points. These genes were clustered into four major categories. The N1 genes were mainly enriched in terms such as serine hydrolase activity and disaccharide metabolic process, indicating that these types of genes are mainly involved in metabolic changes following the stress response; the N2 genes were mainly enriched in lipid transporter activity and transferase activity, indicating that these types of genes are mainly involved in transmembrane transport and other pathways; the N3 genes were mainly enriched in ADP binding and polysaccharide binding, indicating that these genes are mainly involved in energy binding; and the N4 genes were mainly enriched in oxidoreductase activity and single-organism metabolic process, indicating that these genes are mainly involved in redox. These results were similar to previous studies [1,2].

So far, members of the bHLH [28], bZIP [29], MYB [30], C2H2 [31], AP2/ERF [32], NAC [21], and WRKY [33] transcription factor families have been discovered to have roles in salt stress responses in plants. In this study, 522 transcription factors were found among the DEGs; the most abundant transcription factor family was MYB, followed by SNF2, bHLH, WRKY, and other families. These results are consistent with previous reports on the transcription factors involved in plant responses to salt stress, indicating that transcription factors play a key role in the response of *H. hamabo* to salt stress [20]. In addition, the expression patterns of different transcription factor families have temporal and spatial specificity, indicating that transcription factors have very complex regulatory mechanisms.

To further study the mechanism of *H. hamabo* defense against salt stress, we analyzed the 519 co-expressed DEGs. The findings revealed that ion signal transduction and plant hormone signal transduction pathways were critical in *H. hamabo* sensitivity to salt stress. Seven DEGs were mapped to the ion signal transduction pathway, indicating that genes related to osmotic stress in *H. hamabo* can be highly activated after salt stress [34]. Six DEGs were mapped to the ABA signal transduction pathway. These results were consistent with the known ABA regulatory pathway genes, indicating that salt stress can activate the ABA signaling pathway and affect the expression of *PYL, PP2C*, and *SnRK2* genes [22]. Transcription factors are also an important part of the ABA signal transduction pathway. They are usually phosphorylated by protein kinases and directly control the expression of downstream stress-responsive genes [2]. In this study, thirteen DEGs were associated with transcription factors that respond to stress, including three *WRKY*, five *bHLH*, and five *AP2/ERF* genes.

Some *WRKY* family genes in plants, such as *moso bamboo PeWRKY83* and apple *MdWRKY30*, are positively regulated for salt tolerance [35,36]. Salt tolerance is negatively regulated by certain *WRKY* family genes, such as Chrysanthemum *CmWRKY17* and *Polygonum cuspidatum* Sieb. et Zucc. *PcWRKY33* [37,38]. In this study, *HhWRKY79* transgenic *A. thaliana* was significantly more resistant to salt stress than the WT.

## 4. Materials and Methods

### 4.1. Plant Materials and Salt Treatment

Seeds of *H. hamabo* were collected from Nanjing’s Sun Yat-Sen Memorial Botanical Garden and soaked in strong sulfuric acid for 10 min before being grown for 3 weeks in a greenhouse incubator (16 h/8 h light/dark; relative humidity, 65%). Afterward, the seedlings with similar tap root lengths were transferred into 50 ml centrifuge tubes and then cultured in half-strength Murashige and Skoog (1/2 MS) nutrient solution at pH 5.8 for 1 week. The solution was refreshed every 2 days during seedling growth. Then, the seedlings were set into 1/2 MS containing 200 mM NaCl for 48 h or 72 h, which were set as CL48 and CL72, and the control seedlings were cultured in standard 1/2 MS solution (CK). For CK, CL48 and CL72, nine seedlings collected from three triangle bottles were used as three biological replicates, respectively. All samples (roots, approximately 1.5 cm in length) were taken simultaneously for sequencing analysis; the samples were then frozen in liquid nitrogen and deposited at −80 °C.

### 4.2. Iso-Seq Library RNA Preparation, Sequencing, and Analysis

The RNAiso reagent (TaKaRa Biotech Co., Dalian, China) was used to extract total RNA from each sample. Equal amounts of RNA from nine samples (1 μg per sample) were pooled together to form total RNA, and then the SMRT library was prepared using 3 μg total RNA. The Clontech SMARTer^®^ PCR cDNA Synthesis Kit was used to synthesize the first-strand cDNA, then second-strand cDNA was synthesized by large-scale PCR. A Kapa Hifi PCR package (KAPA Biosystems, CA, USA) was used for PCR amplification and cDNA synthesis. SMRTlink 4.0 (http://www.pacb.com/products-and-services/analytical-sofware/smrt-analysis/, accessed on 16 March 2020) software was used to process the above data, and the parameters were set as follows: min_length 300, max_drop_fraction 0.8, no_polish TRUE, min_zscore −9999, min_passes 1, min_predicted_accuracy 0.8, and max_length 15,000.

CD-HIT (version: 4.6.7) [39] was used to cluster the corrected transcript sequences according to 95% similarity, and the parameters were set as follows: -c 0.85, -T 6, -G 0, -aL 0.00, -aS 0.99, and -AS 30. The core conserved gene set of terrestrial plants, namely Eukaryota (version: V1, number of BUSCOs: 429), was selected and BUSCO (version: 3.0.2) was used to evaluate the completeness of the full-length transcriptome sequences.

All full-length transcripts were annotated using seven online libraries, including the NCBI non-redundant protein (NR) database, the Cluster of Orthologous Groups of proteins, Swiss-Prot Knowledgebase, the Kyoto Encyclopedia of Genes and Genomes (KEGG) database, the Eukaryotic Orthologous Groups (KOG) database, the Gene Ontology (GO) database, and the Protein Family (Pfam) database.

### 4.3. RNA-Seq Library RNA Preparation, Sequencing, and Analysis

Total RNA (1.5 μg) of each sample was used for the RNA-seq library construction. Illumina Hiseq2000 (Illumina, San Diego, CA, USA) was used for library sequencing and generating paired-end reads.

A full-length non-chimeric transcript was used as a reference sequence to obtain isoforms after CD-HIT removal of redundancy, and Bowtie2 (version 2.3.4; Parameter: Mismatch 0) software was used to compare the second-generation high-throughput sequencing data with the above reference sequence. RSEM (version: 1.3.1) was used to compare the results and get the number of readcounts compared to each gene for each sample. The FPKM value was converted to a transcripts per million value to obtain the expression level of each isoform.

### 4.4. Differentially Expressed Gene Analysis

Based on the read count in each treatment, differentially expressed genes (DEGs) were assessed via the DEseq2 R package, with q value < 0.05 and |log_2_(Foldchange)| > 1 set as the threshold. Finally, the DEGs shared by the two were used for subsequent analysis.

For functional annotation analyses, we used the GOseq R packages based on Walleniusnon-central hypergeometric distribution to perform GO enrichment analysis on the DEGs, as they can adjust for gene length bias in DEGs. Finally, KEGG enrichment analysis of the DEGs was carried out using KOBAS software. Venn diagrams and heat maps were drawn using TBTools (version: 1.007).

### 4.5. Generation of HhWRKY79 Transgenic A. Thaliana Plants

The ORF sequence of *HhWRKY79* was cloned into the pCAMBIA1305 vector. Then, *HhWRKY79*-1305 was transformed into *Agrobacterium tumefaciens* GV3101, and *A. thaliana* Col-0 was transformed by the inflorescence infection method [40]. The T3 transgenic *A. thaliana* was identified by hygromycin (150 mg/L) selection for subsequent experimental analysis. To treat the *A. thaliana* with salt stress and drought, the seeds of the transgenic *A. thaliana* and Col-0 (WT) were sterilized and cultured on 1/2 MS medium containing 75 mM NaCl. The root length was measured after 8 days of cultivation at 22 °C.

### 4.6. Statistical Analysis

All experiments included three repetitions, and Student’s *t*-test was used to analyze significant differences, which were taken to be *p* < 0.05. Histograms were drawn using Graphpad Prism (v.8.0) software (GraphPad Software, San Diego, CA, USA).

## 5. Conclusions

Overall, by combining SMRT and NGS technologies, we explored the transcriptomic changes in the roots of *H. hamabo*. We also used *A. thaliana* transformation to confirm the function of the *HhWRKY79* gene and discovered that *A. thaliana* overexpression enhanced salt tolerance. These findings provide a complete characterization of gene transcription and facilitate the understanding of the mechanisms of salt tolerance in *H. hamabo*. This study lays a foundation for discovering genes related to the salt tolerance and molecular breeding of *H. hamabo*.

## Figures and Tables

**Table 1:** Summary for the transcriptome data of *H. hamabo* using PacBio.

	Pacbio
Subreads base (G)	9.92
Subreads number	3,287,061
Average subreads length	3017
CCS	274,380
5′-primer	244,651
3′-primer	245,645
Poly-A	206,861
Flnc	177,383
Average flnc read length	3829
Number of isoform	121,091
Number of unigene	94,562
Mean_length	4206
Min_length	161
Max_length	14,547
ExN50 (consensus)	3766
ExN90 (consensus)	7144

## Data Availability

The data have been deposited to the National Center for Biotechnology Information (NCBI) under accession number PRJNA791507.

## Supplementary Materials

The following are available online at https://www.mdpi.com/article/10.3390/ijms23010138/s1.
